# Can Radiotherapy Empower the Host Immune System to Counterattack Neoplastic Cells? A Systematic Review on Tumor Microenvironment Radiomodulation

**DOI:** 10.3390/curroncol29070366

**Published:** 2022-06-30

**Authors:** Federico Iori, Alessio Bruni, Salvatore Cozzi, Patrizia Ciammella, Francesca Di Pressa, Luca Boldrini, Carlo Greco, Valerio Nardone, Viola Salvestrini, Isacco Desideri, Francesca De Felice, Cinzia Iotti

**Affiliations:** 1 Radiation Oncology Unit, Azienda USL-IRCCS di Reggio Emilia, 42124 Reggio Emilia, Italy; salvatore.cozzi@ausl.re.it (S.C.); patrizia.ciammella@ausl.re.it (P.C.); cinzia.iotti@ausl.re.it (C.I.); 2 Radiotherapy Unit, Oncology and Hematology Department, University Hospital of Modena, 41121 Modena, Italy; bruni.alessio@aou.mo.it (A.B.); 297014@studenti.unimore.it (F.D.P.); 3 Radiation Oncology Unit, Dipartimento di Diagnostica per Immagini, Radioterapia Oncologica ed Ematologia, Fondazione Policlinico Universitario “A. Gemelli” IRCCS, 00168 Rome, Italy; luca.boldrini@policlinicogemelli.it; 4 Radiation Oncology, Campus Bio-Medico University, 00128 Rome, Italy; c.greco@unicampus.it; 5 Radiotherapy Unit, Ospedale del Mare, 80147 Naples, Italy; valerio.nardone@unicampania.it; 6 Radiation Oncology, Azienda Ospedaliero-Universitaria Careggi, University of Florence, 50134 Florence, Italy; viola.salvestrini@unifi.it (V.S.); isacco.desideri@unifi.it (I.D.); 7 Department of Radiotherapy, Policlinico Umberto I, Sapienza University of Rome, 00161 Rome, Italy; francesca.defelice@uniroma1.it

**Keywords:** radiotherapy, tumor microenvironment, cold tumors, radiomodulation, immune escape, abscopal effect, combination therapy, tumor mutational burden, host immune system, radioimmunotherapy

## Abstract

Despite the rising evidence in favor of immunotherapy (IT), the treatment of oncological patients affected by so-called “cold tumors” still represents an open issue. Cold tumors are characterized by an immunosuppressive (so-called cold) tumor microenvironment (TME), which favors host immune system suppression, cancer immune-escape, and a worse response to IT. However, the TME is not a static element, but dynamically mutates and can be changed. Radiotherapy (RT) can modulate a cold microenvironment, rendering it better at tumor killing by priming the quiescent host immune system, with a consequent increase in immunotherapy response. The combination of TME radiomodulation and IT could therefore be a strategy for those patients affected by cold tumors, with limited or no response to IT. Thus, this review aims to provide an easy, rapid, and practical overview of how RT could convert the cold TME and why cold tumor radiomodulation could represent an interesting strategy in combination with IT.

## 1. Introduction

RT is a fundamental resource for oncological patients, and recent experience suggests its growing role in widespread disease. RT, in combination with IT, seems to be a promising approach for patients affected by so-called “cold” tumors, which are commonly less respondent to IT [1].

Cold tumors are characterized by an immunosuppressive tumor microenvironment (TME), defined as “cold” TME, and a worse response to IT if compared to the “hot” tumors. Hot tumors present an inflammatory and immunogenic TME (hot TME), where therapies and the host immune adaptive system can more easily and successfully eradicate neoplastic cells [2]. The key role that the host immune system plays in controlling the tumor growth and its diffusion is well documented, and the TME has been proposed as the main factor influencing the immune response against neoplastic cells [3]. Cold TME constitutes a comfortable habitat, where the carcinogenesis process can develop and then spread. However, TME is not a static element, but it dynamically mutates, and it can be modulated, especially by irradiation, since RT can have an immunosuppressive or an immunostimulant effect on TME, depending on dose, schedule, and timing [4].

RT coupled with IT can therefore offer a successful counterattack to cancer immune-escape because of the RT immunomodulating effect, and it may spur an effective and prompt immune response, favoring local tumor control. In addition, a growing amount of evidence suggests how local RT can trigger an immune-mediated regression of unirradiated and distant metastasis-phenomenon called abscopal effect, i.e., tumor control action of secondary lesions distant from irradiated volume in the same patients, especially when combined with IT (e.g., immune checkpoint inhibitors (ICI)) [5,6,7,8,9].

In this systematic review, we focus on cold TME radiomodulation strategies. We then discuss how RT can temporarily reengineer TME and, in association with IT, create a window of action for the host immune system to reject neoplastic cells.

## 2. Materials and Methods

The Preferred Reporting Items for Systematic Reviews and Meta-Analyses (PRISMA) guidelines were followed to construct the search strategy [10]. Our primary aim was to detect and examine available works about the use of RT to modulate a cold TME. To identify the scientific papers specifically addressing the topic of RT-induced immunological modulation of cold TME, we adopted the following bibliographic resource strategy: (“Radiotherapy” [Mesh] OR radiotherapy OR radiation therapy OR radiomodulation) AND (cold tumor*) AND (“Tumor Microenvironment” [Mesh] OR tumor microenvironment OR tumor immunosuppressive microenvironment OR tumor mutation burden OR abscopal effect). We excluded (“Immunotherapy” [Mesh]) as a keyword, as it implied off-topic literature for the purpose of this paper, which is focused on TME dynamic changes due to RT. Only papers in English were considered. No date limitation was adopted for the bibliographic resource, and the last search was carried out in August 2021.

**Figure 1:**
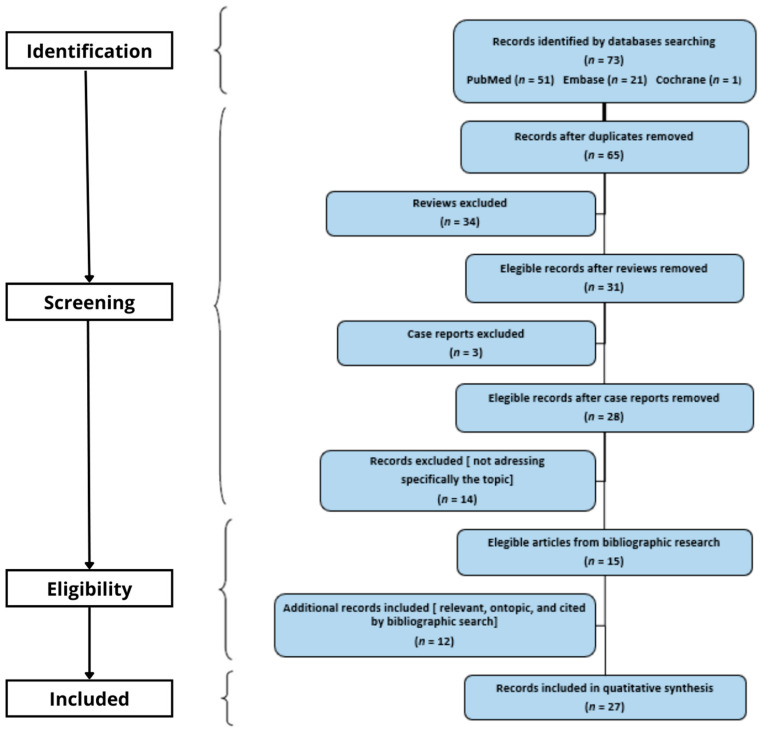
Here we report our Flowchart. The figure displays our flow diagram, i.e., the flow of information through the different phases of our systematic review process, with the records that we found and evaluated in each step.

The database search was performed using PubMed (51 records found), Embase (21 records found), and Cochrane (1 record found). After removing duplicate papers, a total of 65 records were collected, with 34 reviews included. In addition, 12 studies were included as they were relevant for the specific topic and cited in the bibliographic search records found. Regarding the remaining records found through bibliographic research, three case reports were found and excluded. In the end, 27 scientific research papers were considered eligible. In the end, 27 papers were included and evaluated for this systematic review (see PRISMA Flow Diagram in Figure 1). Results were grouped according to the investigated sub-topic. The review was registered on 11 December 2021 in OSF Registries (https://osf.io/svja5, accessed on 10 May 2022). Registration DOI: 10.17605/OSF.IO/SVJA5.

## 3. Results

### 3.1. Tumor Microenvironment

In addition to neoplastic cells, the TME is constituted by local tissue cells, immune cells, and signaling molecules, which are located in a three-dimensional architecture, formed by blood vessels and extracellular matrix [11]. To escape the immune surveillance (“immune-escape” phenomenon), tumors express receptors and signaling molecules that create an immunosuppressive and tolerant TME. The host immune system activation tries to counteract cancer immune-escape by producing an immune adaptive response that generates an immune memory against neoplastic cells. Hence, obtaining a favorable TME may enable an adequate immune reaction against both the primary and metastatic neoplastic sites, potentially achieving a complete disease eradication through in situ vaccination and abscopal effects [12].

### 3.2. Tumor Mutational Burden and Neoantigens Expression

TMB is defined as the number of existing mutations in a megabase of genomic territory. The higher the TMB is, the higher the level of aberrant peptides expressed by cancer cells [13]. Once processed, these peptides act as neoantigens, and they are presented by the major histocompatibility complex of tumor cells, dendritic cells, and APC, priming an effective immune adaptive response against neoplastic cells [14].

### 3.3. Hot and Cold Tumor Microenvironment

TME can be defined as “hot” or “cold” according to its inflammatory and immunologic status. Hot TME is characterized by a high tumor mutational burden (TMB), high neo antigens expression, high type 1 interferon (INF-I) production mainly linked to the STING pathway, high CD8+ T lymphocytes infiltration (TLI), and high dendritic cell (DCs) activation and cross-presentation. Conversely, cold TME is rich in regulatory T cells (Tregs), myeloid-derived suppressor cells (MDSCs), M2 macrophages (M2), and exhausted CD8+, while it presents low CD8+ TLI and TMB with scarce neo antigen expression and presentation [15].

The IFN-I primes the inflammatory cascade activating the immune priming phase and increasing the TLI by recruiting the CD8+ and by promoting the antigens presentation. The higher the TMB, the higher the number of antigens that the host immune cells can target. Once the antigen-presenting cells (APCs)—such as the DCs—have presented cancer antigens, CD8+ plays a key role in the anti-cancer adaptive immune response. This immune cascade is enhanced in hot TME and allows adequate host immune system activation. By contrast, Tregs, M2, and MDSCs are normally involved in the de-escalation of the immune reaction (anti-inflammatory wave), in its ending, and in the maintaining of the immune tolerance to prevent the autoimmunity. Thus, together with the low availability of targetable antigens due to the low TMB, a cold TME is characterized by increased recruitment of these immune elements, which prevents host immune system activation.

### 3.4. Immunosuppression in Cold Tumor Microenvironment

Generally, the immune response consists of two consecutive phases: the priming phase and the effector phase. During the first phase, the irradiation provokes the so-called immunogenic cell death with the release of many damaged associated molecular patterns (DAMPs) as well as many cancer neoantigens. The DAMPs are proinflammatory molecules that contribute to triggering the inflammatory response locally. Conversely, the neoantigens are drained to the closer lymph nodes, where they can be successfully recognized by the APCs. This process starts the acute inflammatory pathways with the release of several inflammatory chemokines and cytokines, promotes the recruitment of effector cells, and prepares the following phase. In the effector phase, the APCs neoantigens cross-presentation generates the immune response against the tumor and, as a result, cancer cells are killed by the immune system. Normally, the anti-inflammatory wave ensues to avoid excessive damage to self-structures, stopping both the inflammatory cascade and the immune system action. However, in cold TME, cancer cells boost the recruitment of Tregs, MDSCs, and M2, which tend to prevent the development of an effective immune reaction by anticipating and boosting the anti-inflammatory phase [16].

Thereby, cold TME neutralizes the immune response onset causing an immune tolerance against neoplastic cells [17,18]. As a result, cold tumors show a limited response to IT because host anti-cancer immunity cannot intervene properly and effectively [19].

Despite cancer immunosuppression and immune escape efforts, a switch into a hot TME means generating acute inflammation features, promoting neoantigen presentation and immunogenic cell death of tumor cells [20]. These TME modifications may favor a better disease response empowering a proper immune adaptive response in combination with IT [21].

### 3.5. The Use of Radiation Therapy to Convert a Cold Tumor Microenvironment into a Hot One

Diverse strategies have been explored to enhance the IT response and increase host immune system activation in cold tumors. Considering that it has been recognized as the key role of TME in tumor immunogenicity and that the TME limited immunogenicity still represents an open issue, the use of RT to reengineer an immunosuppressive TME does represent an appealing option. Evidence suggests that RT can activate or inhibit the immune system according to the RT schedule. RT can provoke immunogenic cell death and increase cancer neoantigen release. In addition, it influences the genetic expression of several molecules involved with the immune response and the leuco-lymphocyte differentiation in proinflammatory or anti-inflammatory verse. Since the cold TME immunosuppressive profile determines a limited efficacy of IT, many studies have investigated how to exploit the proinflammatory and immunogenic effects of RT to improve IT response by switching a cold TME into a hot one [22,23,24].

### 3.6. Cold Tumor Microenvironment Radiomodulation: Preclinical Research

**Figure 2:**
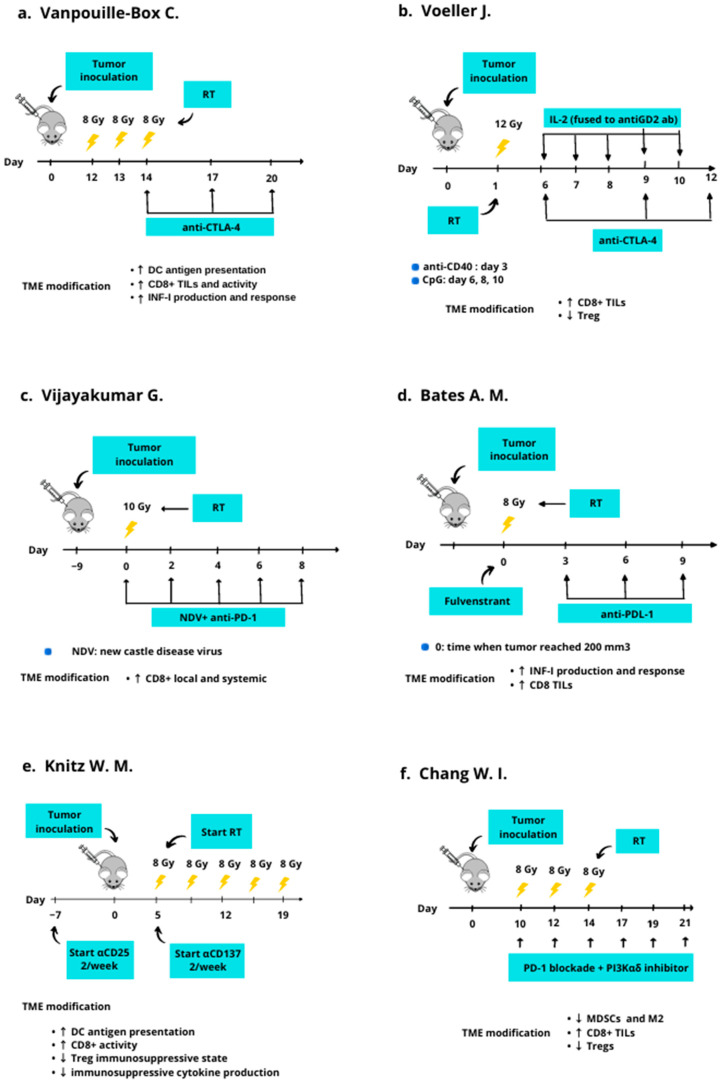
Summary of the experimental protocols associated with the most promising result of preclinical studies of TME radiomodulation and IT. The authors highlighted these combinations as the most effective for switching a cold TME into a hot one, and for increasing tumor response to IT. The figures display the RT schedules (dose/fraction) and the IT tested, as well as the timing of combination. Furthermore, the TME modifications provoked by the treatment are also reported under each figure. (**a**–**f**) The most effective combination of TME radiomodulation and IT tested by Vanpouille-Box et al. [22], Voeller et al. [27], Vijayakumar G. et al. [28], Bates et al. [29], Knitz et al. [30], and Chang et al. [31], respectively.

The discovery of molecular pathways modulated by irradiation, such as the STING pathway and Trex1 exonuclease, has opened new perspectives for TME radiomodulation in the treatment of cold tumors [25,26]. Of the analyzed preclinical works, in Figure 2, we displayed the RT schedules that resulted in the most effective TME radioconversion, higher tumor control, and the best host immune system response in combination with IT; (see Figure 2).

Vanpouille-Box et al. delivered different upfront RT schedules, with or without an ICI, on a mouse mammary carcinoma model to evaluate both local tumor control and abscopal effect (distant tumor control). According to their data, a hypofractionated RT, with 24 Gy delivered in three fractions (8 Gy × three fractions), administered every other day of a week, followed by an ICI (i.e., anti-CTLA-4), resulted in an effective therapeutic scheme to switch the cold mammary carcinoma TME into a hot one (Figure 2a). This upfront radiomodulation schedule associated with IT caused an adequate TME “inflammation” with a marked reduction of both the irradiated and the non-irradiated neoplastic sites [22].

In addition, they observed how this RT schedule could enhance response to IT by activating the downstream STING pathway, a stimulator of interferon genes. The STING activation was promoted by the rise of intra-cytosolic DNA provoked by RT, which boosted inflammatory cytokines expression [19,20]. This process caused an increase in INF-I production, CD8+ activities, CD8+ TLI, and DCs cross-presentation. By contrast, RT dose/fraction above 12–18 Gy appeared to stimulate Trex1 expression, which precluded the STING pathway by degrading intra-cytosolic DNA [22].

Voeller et al. delivered 12 Gy in a single session (i.e., 12 Gy in one fraction) to a mouse-implanted neuroblastoma, a very cold tumor poorly responsive to IT [27]. They evaluated the TME changes and the tumor volume response to this RT schedule, as well as the response changes when different IT combinations were associated with RT. Their data showed that the delivery of 12 Gy induced an initial inflammatory wave in the TME, closely followed by an anti-inflammatory counter-wave, histologically characterized by an increase in Tregs infiltration. To maintain this immunogenic TME and the switch into the hot state, they found that the combination of IT and RT was required. Among the different combinations tested, they highlighted that the administration of 12 Gy combined with an IL-2, anti-CTLA-4, anti-CD40, and CpG (an immunostimulatory nucleotide) (Figure 2b) produced the most positive results, with 80% of the mice showing a complete response after 60 days. In addition, the combination primed the creation of an effective immune memory, which was able to counteract the rechallenge with the same neoplastic cells. According to their data, RT triggered the inflammatory process, IL2 and CpG synergistically corroborated the radiation-induced immune stimulation, while anti-CTLA-4 and anti-CD40 counteracted the anti-inflammatory wave [27].

The authors concluded that 12 Gy in a single shot could produce an inflammatory wave and the TME switch. However, they suggested that a complex combination of immunotherapies was indispensable to neutralize the subsequent anti-inflammatory reaction boosted by cancer cells to escape immune surveillance again [27]. Similarly, Vijayakumar et al. investigated how a different combined strategy of RT and ITs influenced tumor response and the abscopal effect in mice implanted with a B16–F10 melanoma, a well-known cold tumor, with low TMB. Among the therapeutic schemes tested, the upfront delivery of 10 Gy in a single fraction combined with the subsequent administration of an ICI and a virus (Figure 2c) resulted in approximately 90% of complete responses after 60 days from therapy, without significant local adverse effects [28]. Given an intact immune system, RT caused a rise in the proinflammatory and immunogenic features in TME. This produced a conversion from a cold state into a hot one with a peak in local and systemic CD8+ presence. Furthermore, it significantly increased local control. In addition, the synergic action of RT and IT was able to trigger an interesting abscopal response in not-irradiated sites [28].

Vanpouille-Box et al., Vijayakumar et al., and Voller et al. all underlined that the combination of TME radiomodulation and IT could represent a viable strategy against cold tumors by obtaining a favorable and hot TME. However, their works show that the TME switch requires consolidation to enable an adequate and durable immune response [22,27,28].

Preliminary results from Bates and colleagues highlighted that delivering 8 Gy in a single fraction associated with fulvestrant could trigger a valid TME switch and a satisfying response to IT (antiPD-L1) in a cold estrogen receptor positive breast cancer model [29]. More precisely, they concurrently administered fulvestrant (an estrogen receptor antagonist) with radiation treatment of 8 Gy, starting the ICI administration (antiPD-L1) after three days (Figure 2d). Their data underlined that RT could determine a TME switch from a cold state into a hot one, and this conversion was corroborated by concurrent fulvestrant administration. This switch was histologically characterized by an increase in INF-I and in CD8+ TILs in the TME after 10 days. In addition, this TME histological and molecular status opposed immunosuppressive cell trafficking and activation, with an increased response to IT (antiPD-L1) and better tumor control [29].

Knitz et al. evaluated the effect of RT and IT combination in a group of mice with a head and neck squamous cell carcinoma resistant to IT and characterized by a cold TME. Among the different radioimmune schedules tested, the initial administration of an anti-CD25 (interleukin-2 receptor alpha chain), followed by concurrent treatment with RT (40 Gy in five fractions of 8 Gy per fraction) and an anti-CD137- (component of tumor necrosis factor receptor family) (Figure 2e) achieved the most promising result, with 70% of complete responses after 60 days from tumor inoculation [30]. With reference to TME radiomodulation, their data highlighted that hypofractionated RT alone, with 40 Gy delivered in five fractions, could convert the cold TME into a hot one since it induced an increase in antigen presentation, DC recruitment, and CD8+ infiltration and activation. However, this immune system priming was rapidly neutralized by subsequent recruitment of Tregs, which extinguished the RT-induced inflammatory wave. Conversely, when they added the IT combination (an agonist of IL2 and TNF receptors) to RT, they could achieve a more durable TME conversion, with a lower Tregs recruitment and a better response [30].

Chang et al. examined how RT delivery could increase the immunogenicity of a 4T1 murine breast cancer, neoplasia with a very cold TME, and a limited response to IT [31]. They investigated different strategies where RT was delivered alone or associated with IT, and they evaluated the immune-modulatory modification in the TME and the tumor volume response after 31 days from tumor inoculation. In addition, they investigated a potential abscopal effect, considering tumor volume response in the secondary not-irradiated sites. They tested both immune-competent and non-immune-competent mice by delivering a hypofractionated RT with a schedule of 24 Gy in three fractions (8 Gy per fraction delivered every other day), associated or not with different combinations of ITs. Albeit unable to endure, their data showed that RT alone was able to induce an initial conversion of cold TME, with limited control of tumor volume, both in irradiated and not-irradiated sites in the immune-competent mice. Among the tested combinations in the immune-competent mice, the association of hypofractionated RT with a concurrent IT made up of an inhibitor of the PI3K pathway and a PD-1 blockade (Figure 2f) showed a durable TME radioconversion with a response of irradiate lesions and abscopal sites. This latter combination was associated with an increase in CD8+ recruitment and INF-I production, as well as a decrease in Tregs and MDSCs concentration [31]. Besides a well-known immune-suppressive effect, the PI3K pathway modulates Tregs and MDSCs activity and, therefore, it is exploited by cancer cells to neutralize immune surveillance. PI3K is composed of subunits (i.e., alpha, beta, gamma, delta). Interestingly, they observed that the addition of the PI3Kαδ inhibitor reduced Tregs immunosuppressive activity and supported the host immune response, as well as the creation of an immune-adaptive memory against cancer cells [31].

### 3.7. Cold Tumor Microenvironment Radiomodulation: Clinical Research

Albeit the interesting and promising preliminary evidence on cold TME radiomodulation, due to the novelty of the strategy, few clinical data are available about TME radiomodulation in a clinical setting as well as the TME dynamic modifications during the treatment and due to the treatment.

Wilkins et al. retrospectively evaluated how the baseline TME immune status and its radiation induced modification and influenced treatment response in rectal cancers [32]. They analyzed the samples of rectal cancers before and after the patient administration of RT ± chemotherapy. The examined population encompassed patients receiving short-course RT, with 25 Gy in 5 fractions over 5 days (SCRT), and long-course chemoradiation, with the delivery of 45 Gy in 25 fractions over 5 weeks, combined with concomitant fluoropyrimidine-based chemotherapy (LCRT). Patient TME was re-evaluated after the therapy. Interestingly, RT alone or in combination with chemotherapy was able to convert immunosuppressive TMEs into hot TMEs by upregulating genes involved in INF-I production and inflammatory-immune response. By examining the immune gene expression profile and the immune cell/cytokine expression in the TMEs, they highlighted that those patients with a better response or a complete response to the therapy presented a more immunogenic TME at baseline or a more marked TME reprogramming due to the RT ± chemotherapy administration. In addition, their data outlined how those TMEs with a higher level of CD8 TIL and INF-I (typical features of a hot TME) were associated with a better tumor regression [32].

To investigate RT priming action on immune system activity in cold TME, Keam et al. analyzed the TME modification induced by high-dose rate brachytherapy (HDR-BRT) in a group of 24 patients affected by localized prostate cancer, a tumor with a profound immunosuppressive TME. They delivered a schedule of 10 Gy twice 14 days apart, using HDR-BRT [33]. They examined the TME immunological status before and after HDR-BRT delivery using the tumor inflammation signature. Tumor inflammation signature assesses the extent of the upregulation of genes involved in antigen cross-presentation and INF-I production. A higher tumor infiltration signature is associated with an inflamed and immune infiltrate TME and a better response to ICI. Their data showed a radioconversion from a cold TME into a hot one in approximately 80% of the examined tumors. Histologically, this conversion was associated with an increased concentration of APCs, CD8+ lymphocytes, and INF-I. Although this immune status was the expression of the RT-induced initial immunogenic wave, the authors registered subsequent increased recruitment of Tregs that tended to counteract the immune response [33].

## 4. Discussion

Cold TME is a major obstacle for IT response, and, furthermore, it can become a sort of “safe base” for neoplastic cells to develop and spread extensively. Cold TME is an immunosuppressive TME, where neoplastic cells exploit and boost host inflammatory-resolving and immune-suppressing mechanisms to neutralize the host immune system [8]. Since this “immunosuppressive niche” tends to prevent the IT effect, converting a cold TME into a more tumor killing TME (i.e., hot TME) might be an interesting strategy to reactivate the host immune system and increase IT response. RT can trigger an immune response in cold TME according to the schedule delivered, and it can prime an inflammatory wave that tends to reactivate the host immune system and offer a better immune response against cancer cells (TME radiomodulation strategy) [16,34]. As a result, cold TME irradiation might produce an adequate host immune system activation, and it might create an immune memory and circulating effector immune cells capable of rejecting both irradiated and not-irradiated tumor sites (abscopal effect).

In light of this, the identification of an immunogenic RT schedule is mandatory for the TME radiomodulation strategy. The study of Vanpouille-Box and colleagues [22] highlights how a hypofractionated regime (8 Gy × three fractions) can start an immunogenic wave, awakening the host immune system from the tumor-induced quiescence. Their schedule enhances INF-I production by activating the STING pathway without triggering Trex1 exonuclease. Notwithstanding this, adequate irradiated tumor control and abscopal site response were only reached when an ICI was added after RT. This combination maximized the INF-I expression and provided a more durable host immune system activation against neoplastic cells, as also shown by the longer-term ability to control the abscopal sites [22]. Voeller et al. tried to support the radio-induced inflammatory wave and the consequent cold TME radioconversion using a single RT fraction of 12 Gy [27]. However, Vanpouille-Box and colleagues show that at this dose, the Trex1 expression begins to reach concentrations sufficiently to decrease intra-cytosolic DNA, negatively interfering with the STING pathway and consequently reducing the host immune system response [22]. This could explain why Voeller et al. observed a better response in mice that had received the triple combination with RT, IL2-GD2 ab, and anti-CTLA-4. According to their data, complete responses were approximately 40% and 80% for RT alone and triple combination, respectively, after 60 days from treatment start. Interestingly, the combination of ICI and IL-GD2 was associated with a complete response in 10% of cases only. Thus, it might be hypothesized that IL2-GD2 ab strengthened the RT-induced immunogenic-inflammatory wave in cold TME, while anti-CTLA-4 was used to prevent the “anti-inflammatory” wave boosted by cancer cells [27].

Vijayakumar et al. and Bates et al. tested the cold TME radiomodulating effect of single fractions of RT to achieve a more tumor killing TME, and to increase tumor response to IT. They observed more encouraging results in terms of tumor volume reduction and toxicity using a lower RT dose in comparison with Voeller et al. (of 10 Gy and 8 Gy, respectively) [28,29,30].

As with Voeller and colleagues, Vijayakumar et al. highlighted that the addition of New Castle Disease virus as a radioenhancer to RT caused a more marked TME conversion, associated with a local and systemic CD8 recruitment [28]. However, only the ICI administration (anti-PD1) after the virus-RT combination increased the complete responses from 12.5% (NDV virus + RT alone) to nearly 50% (triple combination; Figure 2c) after 80 days from therapy [28].

Since several studies have suggested that estrogen receptor (ER) inhibition negatively influences MDSCs trafficking and activation in TME, Bates et al. tested the combination of an ER antagonist (fulvestrant) and RT (8 Gy × 1), followed by an ICI (Figure 2d). They observed a synergic interaction between RT and fulvestrant with increased recruitment of CD8+ in TME and a better response to the ICI [16,29,30,31,32,33,34]. This strategy might be interesting because RT can induce immunogenic cell death and INF-I production, and it can prime an immune response. However, it has a limited effect on the immune-neutralizing activity of Tregs and MDSC [31]. This might be a reason why Chang and colleagues observed a better result when they tested an RT hypofractionated schedule, like the one used by Vanpouille-Box et al. (8 Gy × 3 fractions delivered every other day), in association with an inhibitor of the PI3K pathway and an ICI [22,23,24,25,26,27,28,29,30,31]. By blocking Tregs′ action with an inhibitor of the PI3K pathway and by supporting the host immune system response primed by RT with the ICI, they achieved a highly marked volume reduction of irradiated and not-irradiated cancer sites in immune-competent mice [31]. Even Knitz et al. highlighted the advantages of using a hypofractionated schedule (40 Gy in five fractions administered on other days; see Figure 2e) to achieve the TME conversion, the host immune system priming, and the tumor volume reduction [30]. In particular, they reported 70% of complete responses when RT priming action and cold TME radio conversion were supported by an enhanced antigen-presenting cell’s activity (anti-CD137) combined with a Tregs block (anti-CD25). This strategy impairs the capacity of neoplastic cells to counteract a new inflammatory/immunogenic response of the host immune system. Notwithstanding this, this strategy could have a point of concern since such a complex combination of IT to support RT priming could be associated with an increased risk of adverse events due to excessive deregulation of the host immune system, with the consequent specter of the autoimmunity risk [35].

**Figure 3:**
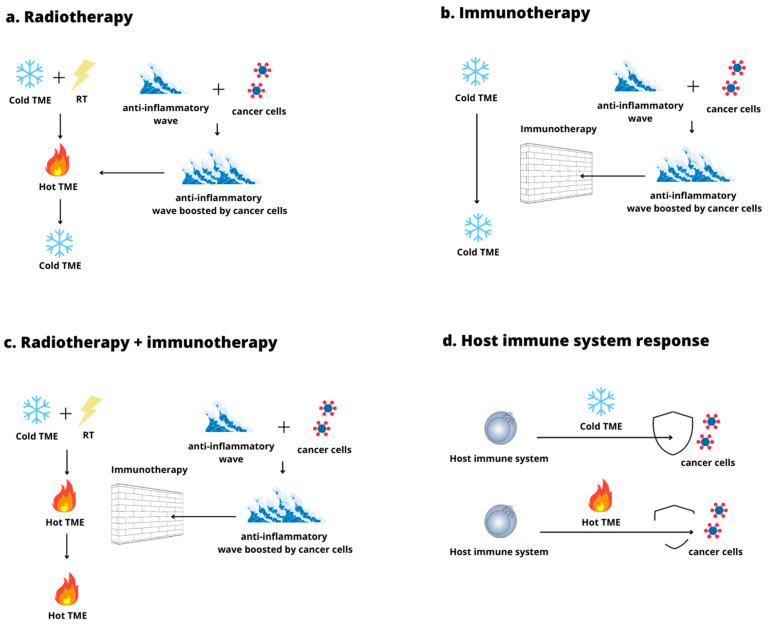
This figure shows how RT alone (**a**) and IT (**b**) cannot trigger a durable TME conversion due to anti-inflammatory wave onset. The anti-inflammatory wave is boosted by neoplastic cells and neutralizes the switch from cold to hot TME, preventing the consequent acute inflammatory and immune response. However, the RT and IT combination enables an effective TME conversion. (**c**) Hot TME is more hostile to cancer cells and provides weaker protection from the immune surveillance and immune response (**d**).

It is interesting to highlight how all the evaluated studies tend to underline the RT capacity to convert cold TME into hot TME by inducing an inflammatory wave. However, this conversion seems not durable alone since the anti-inflammatory and inflammation resolving response, empowered by cancer cells, rapidly follows once RT has triggered the inflammatory cascade in cold TME. Hence, RT can temporarily render cancer cells vulnerable in their niche since it switches the cold TME into a hot state until the inflammation resolving wave arises, reverting the process. Although RT seems to be the key element to temporarily reengineer TME and, consequently, to create the appropriate window of action for the host immune system to strike back, the IT addition appears necessary to render cold TME radioconversion more durable. More precisely, studies tend to suggest that IT is required to thwart the anti-inflammatory wave and allow the host immune response in the brief window of action created by the irradiation. Considering this, due to the switch from cold to hot TME, RT and IT together can allow an effective host immune response against cancer cells (Figure 3) [19,20,21].

Even though Wilkins et al. [32] and Keam et al. [33] showed that RT could convert the TME from a state more favorable to the tumor (cold TME) into a state more favorable to the host immune system (hot TME) with better tumor control, literature suffers the lack of clinical evidence which evaluate this radioimmunological mechanism and the TME modification provoked by the different RT schedules.

Given that TME radiomodulation can increase the response to IT of irradiated and not-irradiated sites, the optimal RT doses and fractions to convert cold TME and prime the host immune system are still under investigation. Due to Trex1, dosage > 10 Gy per fraction seems less promising than 8–10 Gy per fraction administered every other day. Conversely, lower doses per fraction (<8 Gy) appear inadequate to provoke the inflammatory cascade and rearrange the TME. This could be attributed to the tendency of low doses to cause cell apoptosis instead of immunogenic cell death, which is a key point in TME radiomodulation [16]. Thus, current evidence does not allow us to identify a fixed RT schedule (cumulative dose, fractions, and dose/fraction), a precise radioimmune combination (IT choice or IT precise timing), or the right number of lesions that require irradiation in a metastatic setting. Notwithstanding this, available data seem to support a hypofractionated RT schedule with a daily fraction of approximately 8 Gy administered every other day.

Despite limited and initial data and the lack of adequate clinical studies, the available evidence appears to suggest that the TME radiomodulation might be a viable strategy in the management of those cold tumors which are poorly responsive to IT and do not have other therapeutic options. In addition, the TME radiomodulation could be evaluated in the future as a multimodal treatment with curative intent in the metastatic setting, opening a new role for RT in widespread disease

## 5. Concluding Remarks

TME could be considered as a chess board that continuously mutates, influencing the host immune system response in the fight against cancer cells. TME radioconversion could be an important resource to achieve a more favorable battleground (hot TME) and to empower the host immune system, in association with IT, to strike back at neoplastic cells both in the irradiated and not-irradiated neoplastic site. This approach might be interesting and useful in patients affected by cold tumors, and it might open interesting new perspectives for further use of RT in the management of widespread disease.

In addition, a deeper understanding of TME radiomodulation may provide a more robust radiobiological basis for planning a new generation of radioimmune trials. In light of this, further research on irradiation schedules should be undertaken to allow future transfer of TME radiomodulation into the clinical setting.
